# Computational Modeling of Human Mesenchymal Stromal Cell Proliferation and Extra-Cellular Matrix Production in 3D Porous Scaffolds in a Perfusion Bioreactor: The Effect of Growth Factors

**DOI:** 10.3389/fbioe.2020.00376

**Published:** 2020-04-29

**Authors:** Mohammad Mehrian, Toon Lambrechts, Ioannis Papantoniou, Liesbet Geris

**Affiliations:** ^1^Biomechanics Research Unit, GIGA In silico Medicine, University of Liège, Liège, Belgium; ^2^Prometheus, The Division of Skeletal Tissue Engineering, KU Leuven, Leuven, Belgium; ^3^M3-BIORES, KU Leuven, Leuven, Belgium; ^4^Skeletal Biology and Engineering Research Center, KU Leuven, Leuven, Belgium; ^5^Institute of Chemical Engineering Sciences (ICEHT), Foundation for Research and Technology – Hellas (FORTH), Patras, Greece; ^6^Biomechanics Section, KU Leuven, Leuven, Belgium

**Keywords:** computational modeling, mesenchymal stromal cell, perfusion bioreactor, growth factors, optimization, tissue engineering, experimental costs

## Abstract

Stem cell expansion on 3D porous scaffolds cultured in bioreactor systems has been shown to be beneficial for maintenance of the original cell functionality in tissue engineering strategies (TE). However, the production of extracellular matrix (ECM) makes harvesting the progenitor cell population from 3D scaffolds a challenge. Medium composition plays a role in stimulating cell proliferation over extracellular matrix (ECM) production. In this regard, a computational model describing tissue growth inside 3D scaffolds can be a great tool in designing optimal experimental conditions. In this study, a computational model describing cell and ECM growth in a perfusion bioreactor is developed, including a description of the effect of a (generic) growth factor on the biological processes taking place inside the 3D scaffold. In the model, the speed of cell and ECM growth depends on the flow-induced shear stress, curvature and the concentrations of oxygen, glucose, lactate, and growth factor. The effect of the simulated growth factor is to differentially enhance cell proliferation over ECM production. After model calibration with historic in-house data, a multi-objective optimization procedure is executed aiming to minimize the total experimental cost whilst maximizing cell growth during culture. The obtained results indicate there are multiple optimum points for the medium refreshment regime and the initial growth factor concentration where a trade-off is made between the final amount of cells and the culture cost. Finally, the model is applied to experiments reported in the literature studying the effects of perfusion-based cell culture and/or growth factor supplementation on cell expansion. The qualitative similarities between the simulation and experimental results, even in the absence of proper model calibration, reinforces the generic character of the proposed modeling framework. The model proposed in this study can contribute to the cost efficient production of cell-based TE products, ultimately contributing to their affordability and accessibility.

## Introduction

The field of tissue engineering (TE) is constantly evolving but the development of a robust and reproducible tissue engineered advanced therapy medicinal product (ATMP) remains a challenge. Although several studies have shown the potential of TE ATMPs for *in vivo* tissue regeneration (Chai et al., 2012; Roberts et al., 2012), this has been mostly obtained with methods that relied on manual operations. In this respect, bioreactors could play an important role in creating a successful clinical product by contributing in achieving an automated, controlled, and monitored process environment for cell expansion and/or combination product culture (Schneider et al., 2010; Salter et al., 2011). This environment is then amenable to optimization and standardization through the use of *in silico* strategies.

Furthermore, the perfused flow through scaffold pores inside a bioreactor will expose cells to proper mechanical stimuli, which is shown to be beneficial in cell growth and differentiation, as well as ensuring the supply of nutrients such as glucose and removal of metabolic waste such as lactate (Martin et al., 2004; Haycock, 2011). During 3D growth, cells secrete extracellular matrix (ECM) depending on different culture conditions such as the composition of the medium, the frequency of medium refreshment in the bioreactor, the scaffold geometry and the flow rate (Papantoniou et al., 2014b; Sonnaert et al., 2017). Although the presence of ECM has shown to be advantageous for maintaining the potency of the expanded cells (Li and Pei, 2010; Pei et al., 2011), recovering the cells from the 3D scaffold is a challenging procedure. For the purpose of cell expansion in 3D scaffolds, we need to limit the ECM production and increase the cell proliferation. The use of growth factors in the culture medium is a necessity that can significantly increase proliferation or differentiation of cells toward a specific lineage and affect the amount and extracellular matrix that is produced by the differentiating cells (Hankemeier et al., 2005; Rodrigues et al., 2010; Mishra et al., 2016).

Computational models are useful tools in unraveling the complexity involved in neotissue (combination of cells and the extracellular matrix they produce) growth inside 3D scaffolds as they enable us to investigate the effect of a wide range of factors affecting the tissue formation during the culture period, assisting in designing and optimizing the best culture procedure (Lemon et al., 2007; Carlier et al., 2014; Chapman et al., 2014; Misener et al., 2014; Guyot et al., 2015; Shakhawath Hossain et al., 2015; Mehrian et al., 2018).

In a previous study we have developed a computational model describing neotissue growth inside 3D scaffolds in a perfusion bioreactor (Mehrian et al., 2018), taking into account influences of geometry, flow-induced shear stress, oxygen, glucose, lactate, and pH. We have furthermore applied various optimization methods to derive culture conditions leading to maximal filling of the scaffold at minimal cost (Mehrian and Geris, 2020). In contrast to that previous optimization objective, in this study, we do not focus on the optimization of the combination product (scaffold + neotissue) but rather we focus on the use of the perfusion bioreactor set-up to perform 3D cell expansion. Thereto, we have enhanced the previous model by replacing the neotissue variable by two separate variables, one for cell volume and one for ECM volume. The interaction between cell growth and ECM production, as well as the dependence of both variables on external factors, is an intricate process with many quantitative relations currently unquantified (or even uncharacterized). The method presented here provides a framework that can be continuously updated with new information related to a specific biological application in order to increase its biological relevance. In this study, model calibration was carried out based on historic results obtained in our perfusion bioreactor set-up. A (generic) growth factor variable was added to the model, with distinct effects on cell proliferation vs. matrix production. As different input parameters of the model such as refreshment time, refreshment amount and the initial concentration of the growth factor(s) in the medium greatly impact not only the cell and ECM production but also the cost of culture, a multi-objective optimization was run to find the combination of the aforementioned parameters leading to maximum cell volume in the most cost efficient manner. Finally, we have applied the model to two studies reported in the literature where (static or dynamic) cell culture was carried out under the presence of a range of growth factor concentrations. Due to lack of proper calibration information, the comparison remains at the qualitative level. The similarity in trends observed between experimental and simulation results however, further reinforces the generic character of the proposed modeling framework.

## Methods

### Experimental Set-Up

**Figure 1:**
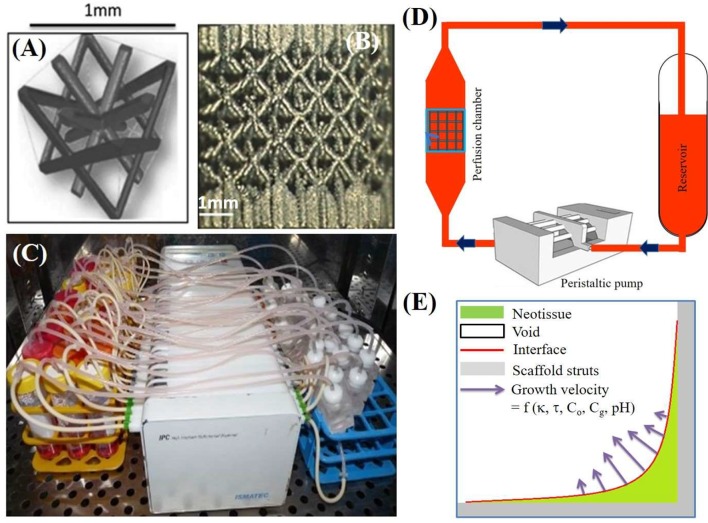
**(A)** The parametric unit cell of the computer-aided design of the porous Ti scaffolds, which consists entirely of identical beams with constant circular cross sections (0.1 mm) and a beam length of 0.9 mm. **(B)** A typical image of a selective laser melting produced Ti scaffold. **(C)** An image of the in-house developed perfusion bioreactor equipped with parallel perfusion circuits. **(D)** Schematic representation of the bioreactor setup used for three-dimensional (3D) dynamic culture, consisting of a medium reservoir containing 10 mL of medium, a peristaltic pump forcing the culture medium through the porous scaffold that was positioned in the perfusion chamber. **(E)** Schematic representation of simulated neotissue growth inside scaffold with growth velocity a function of curvature (κ), flow induced shear stress (τ), oxygen concentration (C_o_), glucose concentration (C_g_), and pH. Adapted from Papantoniou et al. (2014b) and Guyot et al. (2015).

The experiments used in this study have been extensively described in Papantoniou et al. (2014b) and Sonnaert et al. (2017). Briefly, the cells used in this experiment are human Periosteum Derived Cells (hPDCs), chosen for their pluripotency and their bone forming capacity (De Bari et al., 2006). hPDCs were isolated from periosteal biopsies of different donors as described in Eyckmans and Luyten (2006). All procedures were approved by the ethics committee for Human Medical Research (KU Leuven) and explicit patient (or parental) consent was obtained. Cells were expanded in the Dulbecco's modified Eagle's medium with high glucose (Invitrogen) containing 10% fetal bovine serum (BioWhittaker) and 1% antibiotic–antimycotic (100 units/mL penicillin, 100 mg/mL streptomycin, and 0.25 mg/mL amphotericin B; Invitrogen). The seeding density used for the two-dimensional (2D) culture dish hPDC expansion was 6000 cells/cm^2^. hPDCs were passaged at 80–90% confluency. At the time of experiment, cells were trypsinized with Tryple Express (Invitrogen) to be seeded on 3D additive manufactured open porous Ti6Al4V scaffolds (Ø = 6 mm, h = 6 mm, and a diamond unit cell with porosity = 73 ± 1%, strut diameter = 245 ± 2 μm, and pore size = 755 ± 3 μm), produced on an in-house developed selective laser melting machine (Van der Stok et al., 2013) (Figures 1A,B). The obtained TE constructs were cultured in an in-house developed perfusion bioreactor equipped with seven parallel perfusion circuits (Figures 1C,D). Each perfusion chamber, holding a single scaffold, was connected to an individual medium reservoir (disposable 50-mL Falcon tubes; BD Biosciences) containing 10 mL of the cell culture medium via a Tygon (Cole Parmer) tubing and via a two-stop tubing (BPT; Cole Parmer) connected to a peristaltic pump (IPC-24; Ismatec SA). Two different perfusion flow rates were used for the bioreactor culture: the low flow rate used was 0.04 ml/min, while the high flow rate was 4 ml/min. In this study, only the former (low) flow rate was used. Basic Growth Medium in the reservoir was fully refreshed (100%) every 2 days for the entire culture period (not taking into account the volume of medium sitting in the tubing and bioreactor chamber). Filling of the scaffold with neotissue was quantified by means of contrast-enhanced nanofocus computed tomography (CE-nano-CT) as described in Papantoniou et al. (2014b). The DNA content was determined using a highly quantitative and selective DNA assay (Quant-iT™ dsDNA HS kit, Invitrogen) as described in Sonnaert et al. (2017).

### Model Set-Up

In a previous study (Mehrian et al., 2018), we developed a computational model describing the neotissue growth inside 3D scaffolds as a function of several geometrical (see Figure 1E), chemical and physical factors, homogenized in space. In the following, we briefly describe this model, and explain the updated equations describing the evolution of cell volume and ECM volume as separate variables in the model. A detailed description can be found in the Supplementary Material and in Mehrian et al. (2018). An overview of all model variables with their respective symbols is provided in Table 1.

**Table 1:** Overview of all model variables.

**Model variable**	**Meaning**
*V_*n*_*	Neotissue volume
*C_*o*_*	Oxygen concentration
*C_*g*_*	Glucose concentration
C_la_	Lactate concentration
κ	Curvature
pH	pH level
τ	Shear stress
*V*_*ECM*_	Volume fraction of ECM
*V*_*Cell*_	Volume fraction of proliferating cells
*gf*	Growth factor concentration
*p*	Refreshment period
*a*	Fraction of the medium being refreshed

#### Neotissue Volume

(1) $$\begin{array}{r} \frac{dV_{n}}{dt}=A\;f_{s}\left(\tau \right)\;f_{c}\left(\kappa \right)\;h_{1}\left(C_{o}\right)\;h_{2}\left(C_{g}\right)\;h_{3}\left(pH\right)\;\frac{V_{n}}{K_{V_{n}}+\lambda V_{n}} \end{array}$$

Equation (1) expresses the neotissue volume (*V*_*n*_) as a function of the concentrations of oxygen (*C*_*o*_) and glucose (*C*_*g*_), pH level (pH), mean curvature (κ) of the neotissue-void interface inside the 3D scaffold and the shear stress (τ) caused by the medium flow that is perfused through the scaffold as described in Guyot (2015).

The shear stress (τ) influence is incorporated in the model based on Chapman et al. (2014) through (Equation 2) where there exist an optimal shear stress range that enhances the growth (between *a*_1_ and *a*_2_) in the model. High shear stress values (τ ≥ *a*_3_) could be detrimental to tissue growth which results in no growth in our model. The values of the parameters *a*_1_, *a*_2_,and *a*_3_used in this study are shown in Table 2.

(2) $$\begin{array}{r} f_{s}\left(\tau \right)=\{\begin{array}{c} 0.5+\frac{0.5\tau }{a_{1}},\;0\leq \tau <a_{1}\\ \;1,\;a_{1}\;\leq \tau <a_{2}\\ \frac{\tau -a_{2}}{a_{2}-a_{3}},\;a_{2}\leq \tau <a_{3}\\ 0,\;a_{3}\leq \tau \end{array} \end{array}$$

**Table 2:** Overview of all parameter values used in this study.

**Parameter**	**Value**	**References**
β_1_	255	Mehrian et al., 2018
β_2_	3.6*10^5^	Mehrian et al., 2018
γ	0.6716	Mehrian et al., 2018
*a_1_*	0.01	Chapman et al., 2014
*a_2_*	0.03	Chapman et al., 2014
*a_3_*	0.05	Chapman et al., 2014
*V_*o*_*	1.09*10^−17^mol/cell/s	Lambrechts et al., 2014
*V_*g*_*	9.5*10^−17^mol/cell/s	Zhou et al., 2013
*K_*o*_*	1.82*10^−3^mM	Carlier et al., 2014
*K_*g*_*	0.3 mM	Shakhawath Hossain et al., 2015
*ϕ_*cells*_*	2.5*10^13^cells/m^3^	Guyot, 2015
*A*_1_	0.216	This study (GA)
*A*_2_	5.612*10^12^	This study (GA)
λ	1.2836*10^−4^ *s*^−1^	This study (half-life of 1.5 h)
α_1_	0.975 $\frac{ng}{ml}$	This study (GA)
α_2_	12.09	This study (GA)
α_3_	0.1 $\frac{ng}{ml}$	This study (GA)

The function describing the effect of curvature on growth is expressed using a linear function:

(3) $$\begin{array}{r} f_{c}\left(\kappa \right)=\{\begin{array}{c} \kappa ,\;\kappa >0\\ 0,\;\kappa \leq 0 \end{array} \end{array}$$

The influence of oxygen and glucose concentrations on the produced neotissue in Equation (1) is taken into account through the functions *h*_1_ and *h*_2_ where neotissue volume reduces when the species level decreases. Table 2 shows the values of *K*_*o*_ and *K*_*g*_ used in this study.

(4) $$\begin{array}{r} h_{1}\left(C_{o}\right)=\;\frac{C_{o}}{K_{o}+C_{o}} \end{array}$$

(5) $$\begin{array}{r} h_{2}\left(C_{g}\right)=\;\frac{C_{g}}{K_{g}+C_{g}} \end{array}$$

Lactate production in the medium is directly related to the medium pH level and negative influences the neotissue growth. Wuertz et al. (2009) described a detrimental effect of pH on cell fate using (Equation 8) where the neotissue growth rate decreases linearly when the medium pH level decreases.

(6) $$\begin{array}{r} h_{3}\left(pH\right)=\{\begin{array}{c} 1,\;pH>7.1\\ \frac{4}{3}pH-8.5,\;7.1\leq pH<6.375\\ 0,\;pH\leq 6.375 \end{array} \end{array}$$

The supply of nutrients such as oxygen and glucose and the removal of waste product (lactate in our model) in the bioreactor set-up are modeled using (Equations 7–10). In these equations, the right-hand side terms show the production or consumption of the species by the cells, modeled using Michaelis-Menten kinetics with ϕ_*cells*_ being the cell density within the neotissue, *V*_*i*_the consumption rate and *K*_*i*_ the Michaelis-Menten with *i* = *o* for oxygen and *i* = *g* for glucose.

(7) $$\begin{array}{r} \frac{dC_{o}}{dt}=-\beta_{1}\;V_{n}\;\phi_{cells}\;V_{o\;}\;\frac{C_{o}}{K_{o}+\gamma C_{o}} \end{array}$$

(8) $$\begin{array}{r} \frac{dC_{g}}{dt}=-\beta_{2}\;V_{n}\;\phi_{cells}\;V_{g}\;\frac{C_{g}}{K_{g}+C_{g}} \end{array}$$

(9) $$\begin{array}{r} \frac{dC_{la}}{dt}=2\;V_{n}\;\phi_{cells}\;V_{g\;}\;\frac{C_{g}}{K_{g}+C_{g}} \end{array}$$

(10) $$\begin{array}{r} pH=7.4-0.0406C_{la} \end{array}$$

The medium refreshment in the model is simulated by setting the glucose (C_g_) and lactate (C_la_) values to their initial amounts at the requested refreshment time. As in the bioreactor set-up, there is a leakage of oxygen, oxygen value is not reinitialized at each refreshment point. For partial medium refreshment, a percentage weighed average was calculated with the current values of the variables and the medium values. For a more detailed explanation on different model parameters, we refer the reader to the Supplementary Materials or Mehrian et al. (2018).

#### Cell Volume and ECM Volume

At this point, using the current state of the equations we cannot make a distinction between cell and ECM compartments in the neotissue. Given that we want to be able to control cell proliferation vs. matrix production, the variable expressing neotissue volume (*V*_*n*_) in Equation (1) is separated into two variables – one for the cell compartment and one for the ECM compartment, based on Lemon et al. (2007) and shown in Equations (11) and (12).

(11) $$\begin{array}{r} \frac{dV_{ECM}}{dt}=A_{1}\;f_{s}\left(\tau \right)\;f_{c}\left(\kappa \right)\;h_{1}\left(C_{o}\right)\;h_{2}\left(C_{g}\right)\;h_{3}\left(pH\right)\;V_{Cell}\;(V_{T}\\ \;-V_{Cell}-V_{ECM}) \end{array}$$

(12) $$\begin{array}{r} \frac{dV_{Cell}}{dt}=A_{2}\;f_{s}\left(\tau \right)\;f_{c}\left(\kappa \right)\;h_{1}\left(C_{o}\right)\;h_{2}\left(C_{g}\right)\;h_{3}\left(pH\right)\;V_{Cell}\;V_{ECM}\;\\ \;\left(V_{T}-V_{Cell}-V_{ECM}\right) \end{array}$$

The volume fraction of ECM (*V*_*ECM*_) in Equation (11) is not only affected by chemical (e.g., oxygen) and physical (e.g., curvature) factors (see Equation 1), but is also considered to be influenced by the cell volume (*V*_*Cell*_). In addition, the production of ECM is limited by the presence of cells, ECM and the total available space (*V*_*T*_). In Equation (12), the volume fraction of proliferated cells (*V*_*Cell*_) is considered to be proportional to *V*_*ECM*_ to simulate the stimulatory effect of extracellular matrix proteins such as the extra-cellular protein Dickkopf-1 (Dkk-1) on cell proliferation (Gregory et al., 2003; Lemon et al., 2007).

To investigate whether all the chemical and physical factors affecting the neotissue volume (*V*_*n*_) in Equation (1) should remain present in each of the two separated (Equations 11, 12), a literature review was conducted to study the effect of each factor on cell proliferation and matrix production.

Shear stress is believed to enhance cell proliferation and differentiation in 3D scaffolds in the presence of fluid flow (Datta et al., 2006; Stiehler et al., 2009). Using different flow rates, mineralized matrix deposition and cell proliferation is increased compared to the static culture (Bancroft et al., 2002; Papantoniou et al., 2014b). MSC cells are shear sensitive and shear responsive with fluid flow induced shear stress affecting their growth and phenotypic state. However, high shear stresses have been seen to be detrimental to MSCs either due to detachment from the scaffolds or due to mechanical damage (McCoy and O'Brien, 2010). The exact thresholds and parameters to describe these processes are dependent on the cell type. In this study, we perform a model calibration procedure based on historic data obtained for hPDCs during culture in a perfusion bioreactor. For this specific cell type and scaffold geometry we have indeed observed that excessively high shear stress affects local growth of neotissue (Papantoniou et al., 2014a). The shear stress magnitudes observed in that study match those in our computational investigation. In this study, the flow rate is kept at a fixed value of 0.04 ml/min.

Scaffold pore size influences the MSCs proliferations and matrix deposition (Oh et al., 2010; Nava et al., 2016) which is an indication of curvature in Equations (11) and (12). In Matsiko et al. (2014), it is shown that scaffolds with the largest mean pore size (300 μm), will result in higher cell proliferation and matrix deposition. In a recent review on curvature topography, Callens et al. (2019) summarize the evidence demonstrating that curvature is driving neotissue formation in a 3D context (which is the baseline assumption of the computational framework).

There are numerous studies showing the effect of oxygen on cell proliferation and differentiation (Choi et al., 2014; Atashi et al., 2015). In Grayson et al. (2006), hMSCs were cultured under two different oxygen conditions (normoxic 20% and hypoxic 2%). Differentiation and proliferation of cells was reported to be higher under hypoxic conditions.

Glucose is the main nutrient for cell growth in our model, but high glucose concentrations could suppress cell proliferation as it is shown in Kato et al. (2016). In this paper, authors compared cell proliferation and differentiation in four different concentrations of glucose (from 5.5 to 24 mM), where the lowest glucose concentration resulted in the best outcome.

In Singh (2014), the author has shown the influence of pH level on cell proliferation and differentiation by comparing the viability of staining MSCs cultured in medium with different pH levels at 21% oxygen where an increased presence of dead cells at lower pH levels was observed. Additionally, the effect of pH on cell proliferation and differentiation could be derived indirectly from the effect of glucose on cells in Equations (8)–(10).

In order to compare model output and experimental results, the experimentally DNA content (Sonnaert et al., 2017) was converted into an indication of the amount of cells and further into a volume measure by multiplying the amount of cells by the typical volume of a single hMSC, taken to be of spherical shape with a diameter of 20 μm (Lemon et al., 2007). Using this hypothesis, the volume of each hMSC will be 4.2^*^10^3^*um*^3^. In Docheva et al. (2008), the authors measured the volume of each hMSC by atomic force microscopy on fibrous substrates (polystyrene and collagen I) and glass. Taking the average volume of hMSCs on these substrates results in a volume of 4.16^*^10^3^*um*^3^ for each hMSC, which is similar to the previous method. The volume fraction of the cells is obtained by dividing the cell volume to the total available space of the scaffold (*V*_*T*_).

#### Growth Factor

Making a distinction between cells and ECM in the model enables us to add a (generic) growth factor to the model equations with a differential effect on proliferation of cells vs. ECM production as shown in Equations (11) and (12). In this study, given the application in cell expansion, the described effect of the growth factor is to enhance cell proliferation and limit the ECM production.

(13) $$\begin{array}{rc} \frac{dV_{ECM}}{dt} & =A_{1}\;f_{s}\left(\tau \right)\;f_{c}\left(\kappa \right)\;h_{1}\left(C_{o}\right)\;h_{2}\left(C_{g}\right)\;h_{3}\left(pH\right)\;V_{Cell}\\ \;\left(V_{T}-V_{Cell}-V_{ECM}\right)\frac{\alpha_{1}}{1+gf} \end{array}$$

(14) $$\begin{array}{rc} \frac{dV_{Cell}}{dt} & =A_{2}\;f_{s}\left(\tau \right)\;f_{c}\left(\kappa \right)\;h_{1}\left(C_{o}\right)\;h_{2}\left(C_{g}\right)\;h_{3}\left(pH\right)\;V_{Cell}\;V_{ECM}\\ \;\left(V_{T}-V_{Cell}-V_{ECM}\right)\frac{\alpha_{2}\cdot gf}{\alpha_{3}+gf} \end{array}$$

(15) $$\begin{array}{rc} \frac{dgf}{dt} & =-\lambda \;gf \end{array}$$

The effect of growth factor is incorporated in Equations (13) and (14). Degradation of growth factor is expressed in Equation (15). Parameter λ is calculated based on the half-life of the growth factor which is in the range of a few hours for the growth factors that are typically used in the context of cell expansion. In this model, we have assumed the half-life of the growth factor to be 1.5 h. Parameters *A*_1_, *A*_2_, α_1_, α_2_, *and α*_3_ are obtained using a genetic algorithm procedure explained in the following sections.

### Model Implementation, Calibration, and Optimization

#### Model Implementation

The model developed in this study was composed of six model variables (*C*_*o*_*, C*_*g*_*, C*_*la*_*, V*_*ECM*_*, V*_*CELL*_*, gf* ) and implemented in MATLAB®. The initial concentration of different model species are as follows; glucose: $25\;\frac{mol}{m^{3}}$, oxygen: $0.192\;\frac{mol}{m^{3}},$ and lactate is zero. The initial cell volume is 1.39% of the available space of the scaffold, which corresponds to the 10^5^ initial seeded cells onto the scaffold. The initial ECM volume is zero. For the initial value of the growth factor, we have assumed that the baseline value for growth factor concentration in the medium corresponds to 1 $\frac{ng}{ml}$.

#### Model Calibration

In order to find the best set of model parameters (*A*_1_, *A*_2_, α_1_, α_2_, *and α*_3_) resulting in the closest model outcome to experimental data, a genetic algorithm was used with the goal to minimize the distance between experimental values (red dots) and model predictions at certain time points. The values of all model parameters are shown in Table 2.

#### Model Optimization: Cost Function

The goal in multi-objective optimization (MOO) is to reach a compromise between several conflicting objectives, in the context of this study that would be to maximize the cell volume whilst minimizing the cost. For solving the MOO problem we have used Particle swarm optimization (PSO) technique which is a recent approach inspired by the choreography of a bird flock. PSO was first introduced by Kennedy and Eberhart (1995) and has been found to be successful in a wide variety of optimization tasks (Kennedy, 2006). In order to find the best answer to the problem, a random population of candidates called “particles” are created. Each particle moves in the search space based on its position and velocity following a mathematical formula. The movement of the particles is influenced by the local best and global best-known positions by the total population and the velocity of each particle updates according to its distance from the best-known positions. This process for finding the best answer is repeated until the population converges or the algorithm reaches the maximum (pre-defined) number of iterations. In this study, the optimization problem was run using 100 initial candidates and stopped after 50 iterations. In order to reach the best answer to the problem, the Pareto frontier (Horn, 1997) is calculated. Pareto optimality is a state in which it is impossible to improve the value of one objective function without worsening the value of the other. In other words, we are looking for the border between the infeasible and the suboptimal in our problem.

The function that we aim to minimize in our MOO problem describes the associated costs of labor and culture medium, including the growth factor, and is expressed in Equation (16).

(16) $$\begin{array}{r} C=\left(M+Gf_{i}\left(P_{g}\right)\right)\;\left(1+\frac{24d}{p}a\right)+\left(L\right)\;\left(1+\frac{24d}{p}\right) \end{array}$$

In this equation, C is the total cost of the experiment, *M* is the cost of the medium used for one medium exchange that is 0.2611€ and *L* is the labor costs for one medium refreshment that is 6.8€. *Gf*_*i*_ is the initial concentration of the growth factor, *P*_*g*_ is the price for 10ml (the reservoir capacity of the bioreactor) of the used growth factor, *d* is the total days in which the experiment lasts, *p* is the refreshment period and *a* is the fraction of the medium being refreshed each time **(**0 ≤ *a* ≤ 1).

### Model Validation Using Experimental Data

We have compared the outcome of the model developed in this study with two other experimental studies looking into the (combined) effects of perfusion and growth factors dosing on the proliferation of MSC-type of cells. In Eom et al. (2014), the authors studied the effect of four different growth factors (FGF-2, FGF-4, EGF, and HGF) on the proliferation of the bone marrow-derived mesenchymal stem cells (BMSCs) derived from three healthy donors (aged 21–40 years) in a dose dependent manner (1, 5, and 10 ng/ml) for 3 days. The cells were cultured in 75 cm^2^ flasks and the culture medium was changed twice weekly. In another study Koller et al. (1993) used hematopoietic stem cells cultured in a 2D+ perfusion environment with the cytokine concentration of 1.5 ng/ml and the culture medium was changed every 5 days by 50%. Due to lack of experimental data, proper model calibration for the aforementioned specific set-ups is not feasible. Therefore, simulations were conducted using the model optimized for 3D perfusion-based culture with hPDCs. As a result, only qualitative comparisons are made.

## Results

### Model Calibration Using Experimental Data

**Figure 2:**
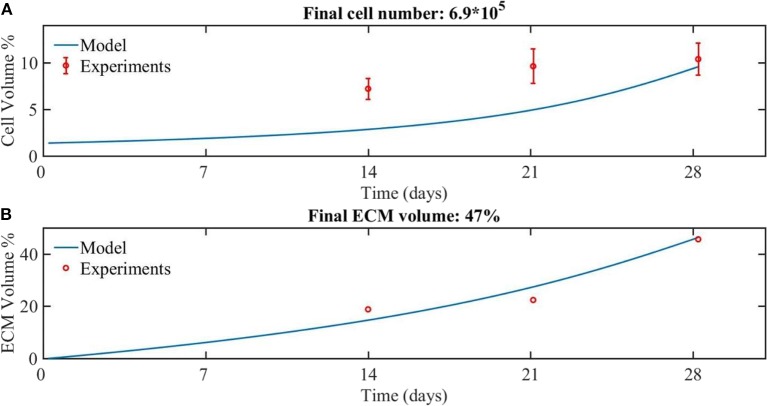
The experimental (red dots) and modeling (blue line) results for the growth of **(A)** cells and **(B)** ECM volume over 28 days of culture. The amount of cells was experimentally estimated based on DNA quantification (Sonnaert et al., 2017). Results are shown as mean ± standard deviation. The ECM volume was measured by contrast-enhance nanofocus CT imaging (Papantoniou et al., 2014b).

The model is calibrated using the experimental data with a flow rate of 0.04 ml/min for the cell and ECM compartments (Figure 2). In the experiments, the medium was refreshed every 2 days by 100% during 28 days of culture. In order to be able to consider the effect of growth factor in the experimental data, it is assumed that in the culture medium the concentration of the growth factor was at its baseline level of 1.

Comparing the numerical and experimental cell volume (Figure 2A), the numerical results (continuous line) showed a longer lag phase compared to the experimental data (dots), but the final cell volume was similar to the experimental data. The corresponding cell number at day 28 was calculated based on the cell volume, being 6.9^*^10^5^ cells. The experimental values obtained from the nanoCT imaging included the cell compartment as well as the ECM compartment. Therefore, for the sake of comparison with model outcome, the experimentally estimated cell volume was subtracted from the experimentally measured total volume to obtain the ECM volume. A good correspondence is obtained between numerical and experimental results for the ECM volume (Figure 2B).

### Model Predictions for Cell and ECM Volume

**Figure 3:**
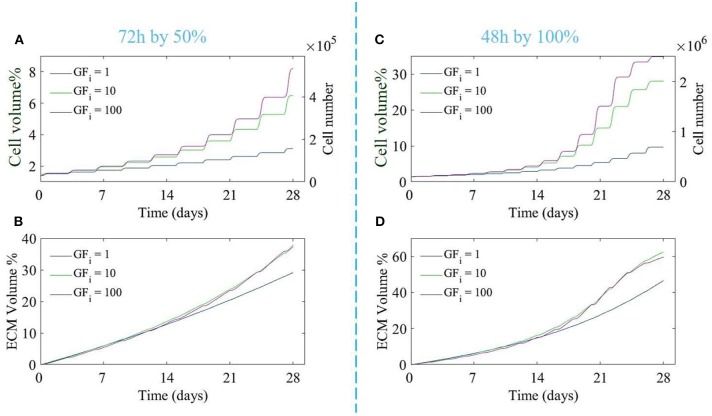
The proliferation of cells **(A,C)** and production of ECM **(B,D)** over 28 days of culture for different initial growth factor concentrations of 1 (blue line), 10 (green line), and 100 (magenta line), and for two different medium refreshments regimes: **(A,B)** medium refreshed every 72 h by 50%, and **(C,D)** medium refreshed every 48 h by 100%. The cell growth is shown by the cell volume % on the left axis and the equivalent cell number on the right axis **(A,C)**.

In order to investigate the effect of different doses of the growth factor and the medium refreshment regime on the cell and ECM production, six different cases were considered, being 3 growth factor concentrations, the baseline concentration of growth factors, and 10 and 100 times the baseline concentration, and two medium refreshment regimes, every 48 h by 100% or every 72 h by 50%. The results are shown in Figure 3.

Both the growth factor concentration and the refreshment regime influence the growth of the cell and ECM compartments. For the case where the medium was changed every 72 h by 50%, increasing the dose of growth factor did not result in a substantial increase in cell or ECM production (Figures 3A,B), especially for higher doses of growth factor (10 and 100). For the case where the medium was refreshed every 48 h by 100%, ECM production was not much effected by the increase in growth factor concentrations whereas the difference in cell volume using different concentrations of growth factor was noticeable. Increasing the growth factor concentration from 10 to 100 did not result in a strong increase in cell proliferation due to the saturation effect (Equation 14). In order to find the best refreshment time and amount for the medium exchange during the culture period as well as the best concentration of the growth factor, a Multi-Objective-Optimization (MOO) problem was solved using Particle Swarm Optimization (PSO) with the goal to minimize all associated costs explained in Equation (16) and maximize the cell proliferation in the scaffold.

### Multi-Objective Optimization

**Figure 4:**
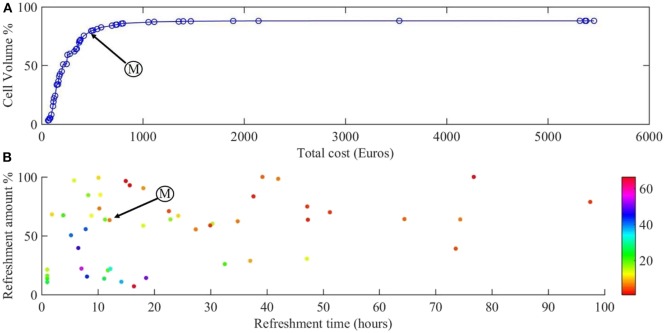
Results of the multi-objective optimization. **(A)** Pareto front for the cell volume and total cost of the experiment. **(B)** The refreshment time and refreshment amounts corresponding to the points on the Pareto front, with growth factor concentration indicated in color. Point M is the sweet spot on the Pareto front where the best compromise between cost of experiment and scaffold filling percentage is obtained.

Figure 4 shows the Pareto front for the two objectives of our problem: cell volume and total cost.

Figure 4A shows the Pareto front for maximizing the cell number during 28 days of culture and Figure 4B the corresponding refreshment time, refreshment amount, and the initial concentration of growth factor (colors). All the points on the calculated Pareto front are considered as optimum points but there exists a single point which is known as the sweet spot on the Pareto front (Figure 4A, point M), where a good compromise between cost of experiment and scaffold filling percentage is obtained. At this point, around 80% of the scaffold is filled by the cells with the cost of 492€ which corresponds to refreshing the medium every 12 h by 65% with the growth factor concentration of 7 (Figure 4B, point M). Moving from this point toward the right-hand side of the Pareto front will result in slightly higher cell numbers (5% more cells), but at a considerably higher culture cost as this regime would require more frequent medium refreshments and higher concentrations of growth factor. Most of the expensive solutions are shown in Figure 4B in the bottom-left corner where the frequency of medium refreshment is very high (<15 h) and the concentration of the growth factor is the highest (light colors) compared to other solutions. For example, it is proposed that if we refresh the medium every 7 h by 40% using 50 times of the initial concentration of growth factor, the final cell volume would be around 87% with a total calculated cost of 1,411€ whereas we can reach 80% of cell volume with a total calculated cost of 500€.

### Comparison With Other Experimental Studies

**Figure 5:**
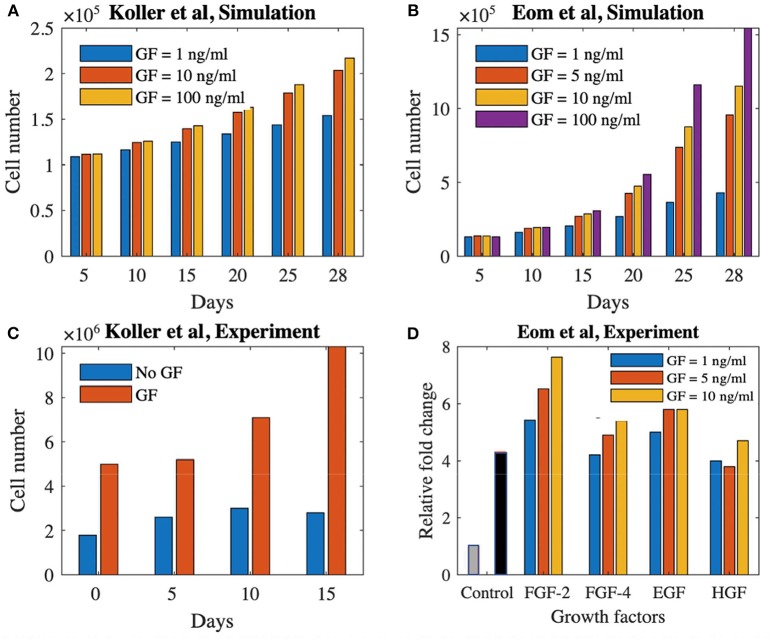
Proliferation potential of cells treated with growth factors in static and dynamics culture set-ups. **(A)** Simulation result of cell proliferation (Koller et al., 1993) using three different concentrations of a growth factor. **(B)** Cell proliferation with and without growth factor for the experimental study of Koller et al. (1993). **(C)** Simulation result of cell proliferation (Eom et al., 2014) using four different concentrations of a generic growth factor. **(D)** Cell proliferation using four different growth factors with three different concentrations at day 3 (Eom et al., 2014). The gray and black bars shows the control group (no growth factor) at day 0 and 3, respectively. GF, growth factor.

Figure 5 shows the comparison between our model predictions and the two aforementioned experimental studies (Koller et al., 1993; Eom et al., 2014). Figure 5A shows simulation results for the Koller et al. set-up (medium refreshment every 120 h by 50%, no flow), focusing on cell number increase over 28 days of culture using different growth factor concentrations (1, 10, and 100 ng/ml). Figure 5B shows the experimental data from Koller et al. (1993) where the cell proliferation is shown with and without growth factors during 15 days of culture time. Despite obvious quantitative differences (in time and amount of cells) owing to lack of model calibration, the same qualitative trends are visible.

Figure 5C shows the simulation results for the Eom et al. study where four different concentrations of growth factor (1, 5, 10, and 100 ng/ml) are used during 28 days of culture time in a 2D+ perfusion set-up (medium is refreshed every 55 h by 100%). The experimental results of Eom et al. are shown in Figure 5D where the relative fold change in cell proliferation for the four different growth factors up until days 3 is presented compared to static controls. Again, in absence of proper calibration, only qualitative comparison is possible, showing similar trends in cell proliferation for different growth factors doses between our simulations and the experimental data.

## Discussion

In this study we have further extended a previous model describing neotissue growth inside 3D scaffolds. Given that here the intended use of the bioreactor set-up and associated model was situated in the context of cell expansion, rather than the production of neotissue, separate equations have been developed to describe the cell and ECM volume. Subsequently, the effect of a generic growth factor has been incorporated in the model, allowing for a differential stimulation of cell proliferation over ECM production. Using this model, a multi-objective optimization strategy has been implemented, using PSO, with the aim of maximizing cell number while minimizing the corresponding experimental costs. The calculated Pareto front proposed multiple optimum points that we can choose from, depending on the desired cell number and its associated cost. Finally, a qualitative comparison was made with experimental results reported in the literature, showing qualitative similarities despite obvious quantitative differences due to absence of proper calibration which reinforces the generic character the of proposed modeling platform. This study provides an *in silico* framework that, when calibrated for a particular cell type of interest, allows to identify meaningful culture regimen optima. This can provide an important support to a decision-making process that is currently mainly empirical.

The developed model in this study could be used to predict the cell number in 3D scaffolds cultured in a bioreactor set-up. One of the main issues accompanied with using 3D scaffolds for the purpose of cell expansion is the recovery of the cells from the scaffold at the end of culture, a process that is still largely an under-investigated field (Abbasalizadeh and Baharvand, 2013; dos Santos et al., 2013). In the perfusion set-up used in this study, Sonnaert et al. (2015) tested three different reagents to release the cells from the 3D culture surface and the best outcome was obtained using the collagenase reagent where 76% of the cells were recovered from the scaffold. They set the time point for cell recovery at 13 days to prevent over-confluence. This time point was chosen with respect to the metabolic activity measurements which is a frequently used measure for the amount of proliferated cells inside the scaffold. The model presented in this paper (after complete validation with dedicated experimental studies) will enable us to track cell growth during the whole culture period and therefore, to have a better estimate on the best cell recovery moment.

Notwithstanding the issues surrounding the recovery of cells from the 3D substrate, cell expansion in 3D scaffolds has many advantages compared to conventional 2D cultures. In 2D culture flasks, the cell expansion procedure becomes more labor intensive for each additional passage until the required amount of cells is reached. Whereas in the 3D expansion process, multiple scaffolds can be cultured in parallel with minimum interference from the operator. Additionally, 3D cell culture will result in a more robust and reliable cell expansion process. In Papadimitropoulos et al. (2014), the authors compared the MSC expansion in 2D flasks and 3D scaffolds and observed a similar proliferation capacity in the two methods, although a 4.3-fold higher clonogenicity capacity and a higher differentiation capacity toward all lineages was reported in 3D culture. Furthermore, in Lambrechts et al. (2016), authors compared the 2D with 3D cell culture and they observed a 2.5 lower variability on cell yield (normalized by culture surface) for the bioreactor culture compared to the flask-based expansion. Therefore, in time, 3D cell culture could replace most of the 2D cell culture. In this regard, the model developed in this study is a step forward in moving from manual tissue engineering strategies toward a more integrated and automated solution for expanding stem cells by providing an appropriate tool that predicts the cell proliferation during culture time. Furthermore, the proposed optimization strategy in this study would minimize the use of growth factors as they are one of the main sources of cost in the experiments and therefore, brings us one step closer in development of an affordable tissue engineered ATMP. In this study we have taken the current commercial price of typical growth factors used in a TE context (BMP-2). In the future it can be replaced with any other growth factor which enhances the cell proliferation over cell differentiation. One of the main limitations in this study is that due to the lack of experimental data, we were unable to fully validate the final model for the early time points as well as for the equations related to the incorporation of growth factors, which therefore remain mostly conceptual.

In terms of verification and validation of the model, all necessary verification steps have been executed and described in our previously published body of work (Guyot et al., 2015; Mehrian et al., 2018). As to validation of the model, we have used input from different studies to either qualitatively or quantitatively validate specific model predictions. As to the relative volumes of cells and ECM, we have partially validated the model with pre-existing experimental data (Papantoniou et al., 2014b), where we assumed the concentration of the growth factor at its baseline level of 1 in the culture medium. Adding growth factors during culture increases the proliferation potential (Figures 3, 5A,C). The saturation of the growth factor effect that was observed in the simulations has been reported in Mishra et al. (2016), where the authors showed that increasing the growth factor dosage by 10-fold did not significantly increase the proliferation rate of the cells. Also Eom et al. (2014; Figure 5D) showed that increasing the growth factor dose does not increase the proliferation capacity in a linear manner. Despite qualitative correspondence, there are substantial qualitative difference in both time and cell volume between the simulations and experimental results shown in Figure 5. Due to lack of data regarding several aspects of the experimental set-ups used by Koller et al. (1993) and Eom et al. (2014), model calibration could not be carried out. Several set-up specific elements explain the observed quantitative differences. In the study by Koller et al. (1993) hematopoietic stem cells were used, which are quite different from the hPDCs used in our model calibration experiments. Furthermore, these hematopoietic stem cells were cultured under perfusion on a 2D+ substrate made of bone marrow stroma (layer-substrate) which is quite a different environment form the 3D scaffold and neotissue environment provided to the hPDCs in our set-up. Given these differences, it is encouraging that the model is able to qualitatively capture the growth factor influenced cell growth even for different types of cells. The cell type used in Eom et al. (2014) is more similar to our study, however the culture set-up (static, 2D culture plastic) and medium are different. This leads to a quantitative difference (temporal behavior and amount of cells) whilst showing a qualitative agreement between simulation results (Figure 5C) and experimental results (Figure 5D) especially for the FGF-2 growth factor. These results are in line with the model predictions presented in this study (Figure 2) where we show that there exists a saturation level (as it is implemented in the equations) for the effect of the growth factors on the proliferation of the MSC–type of cells. In order to go from these conceptual qualitative demonstrations toward tangible quantitative predictions for specific cell sources, model parameters will need to be calibrated with appropriate detailed data from dedicated experiments.

In conclusion, we have developed a computational model describing the cell and ECM production inside 3D scaffolds during perfusion bioreactor culture and we have optimized the performance of the model resulting in maximum cell number minimizing the associated costs of experiment. The developed model in this study could contribute in the trend moving from 2D cell cultures to a more promising cell expansion process in 3D environment.

## Data Availability Statement

The datasets generated for this study are available on request to the corresponding author.

## Ethics Statement

The studies involving human participants were reviewed and approved by Ethics committee for Human Medical Research (KU Leuven). The patients/participants provided their written informed consent to participate in this study.

## Author Contributions

MM developed the computational model and wrote the manuscript. TL, IP, and LG contributed to the interpretation of the results. LG conceived and supervised the overall project. All authors commented on the manuscript.

## Conflict of Interest

The authors declare that the research was conducted in the absence of any commercial or financial relationships that could be construed as a potential conflict of interest.
