# Supplementary material for: Computational Modeling of Human Mesenchymal Stromal Cell Proliferation and Extra-Cellular Matrix Production in 3D Porous Scaffolds in a Perfusion Bioreactor: The Effect of Growth Factors
Source: Front Bioeng Biotechnol. 2020 Apr 29;8:376. doi: 10.3389/fbioe.2020.00376 (PMC7201129; doi:10.3389/fbioe.2020.00376)
Supplement: Supplementary file 1 [file Data_Sheet_1.DOCX]

**Supplementary methods**

This document corresponds to our paper: ‘Computational modelling of human mesenchymal stromal cell proliferation and extra-cellular matrix production in 3D porous scaffolds in a perfusion bioreactor: The effect of growth factors’.

This supplementary material describes the *in silico* ordinary differential equations (ODE) model of neotissue growth on a three-dimensional scaffold in a bioreactor setting. As described in this supplement, the model is already available in the literature (Mehrian et al. 2017). However, we additionally provide this supplement for easier interpretation.

**Mathematical model**

**Neotissue volume**

The neotissue volume produced inside the scaffold is a function of several geometrical, chemical and physical factors.

| $\frac{{dV}_{n}}{dt}=A . f_{s}\left( \tau\right) . f_{c}\left( \kappa\right) . h_{1}\left( C_{o} \right). h_{2}\left( C_{g} \right). h_{3}\left( pH \right). \frac{V_{n}}{K_{V_{n}}+\lambda V_{n}}$ | (1) |
| --- | --- |

Equation (1) expresses the neotissue volume (*V_n_*) as a function of oxygen (*C_o_*), glucose (*C_g_*), pH level (pH), mean curvature (κ) of the neotissue-void interface inside the 3D scaffold and the shear stress ( caused by the medium flow that is perfused through the scaffold as described in Guyot (2015).

As neither flow-induced shear stress nor mean curvature can be calculated in a spatially homogenized model, relationships between these variables and the neotissue volume filling (*nf%*) were derived from the mechanistic heterogeneous model developed by Guyot et al. (2016). The relationships for the shear stress and curvature have been implemented in the model using the following equations:

| $\tau=\left\{ \begin{aligned} s_{1}V_{n}, &nf\%<T_{1} \\ s_{2}V_{n} , &nf\%\geq T_{1} \end{aligned} \right.$ | (2) |
| --- | --- |
| $\kappa=\left\{ \begin{aligned} c_{1}V_{n} , &nf\%<T_{2} \\ c_{2}V_{n} , &nf\%\geq T_{2} \end{aligned} \right.$ | (3) |

Parameters *T_i_*, *s_i_* and *c_i_* are determined through a fitting procedure using genetic algorithms. *T_1_* and *T_2_* are the two turning points for the shear stress and curvature respectively.

For the shear stress influence, Chapman et al. (2014) introduce a growth model for cell aggregates in hollow fiber bioreactors where cell population growth varies depending on wall shear stresses experienced by cells. These findings have been incorporated in Guyot (2015) through Eq. (4) and transferred to the model.

| $f_{s}\left( \tau\right)=\left\{ \begin{aligned} 0.5+\frac{0.5\tau}{a_{1}}, 0\leq\tau<a_{1} \\ 1 , a_{1} \leq\tau<a_{2} \\ {\frac{\tau-a_{2}}{a-a_{3}}, a_{2}\leq\tau<a_{3} \atop0, a_{3}\leq\tau} \end{aligned} \right.$ | (4) |
| --- | --- |

The function describing the effect of curvature on growth is expressed using a linear function:

| $f_{c}\left( \kappa\right)=\left\{ \begin{aligned} \kappa, \kappa>0 \\ 0, \kappa\leq0 \end{aligned} \right.$. | (5) |
| --- | --- |

The influence of oxygen and glucose concentrations on the produced neotissue in Eq. (1) is taken into account through the functions *h_1_* and *h_2_* where neotissue volume reduces when the species level decreases.

| $h_{1}\left( C_{o} \right)= \frac{C_{o}}{K_{o}+C_{o}}$ | (6) |
| --- | --- |
| $h_{2}\left( C_{g} \right)= \frac{C_{g}}{K_{g}+C_{g}}$ | (7) |

Lactate production in the medium is in direct relation with the medium pH level and has a negative influence on the neotissue growth. In Wuertz et al. (2009) a detrimental effect of pH on cell fate was described using Eq. (8) where the neotissue growth rate decreases linearly when the medium pH level decreases.

| $h_{\boldsymbol{3}}\left( pH \right)=\left\{ \begin{aligned} 1, pH>7.1 \\ \frac{4}{3}pH-8.5, 7.1\leq pH<6.375 \\ 0, pH\leq6.375 \end{aligned} \right.$ | (8) |
| --- | --- |

**Nutrients and waste transport**

The influence of model variables such as oxygen, glucose and remove metabolic waste (lactate) on neotissue growth is described in Eq. (9)-(11). In these equations, the right-hand side terms show the production or consumption of the species by the cells, modeled using Michaelis-Menten kinetics with *φ_cells_* being the cell density within the neotissue, *V_i_* the consumption rate and *K_i_* the Michaelis-Menten constants for different species. Parameters *  γ *and *λ* have been determined using a genetic algorithm.

| $\frac{dC_{o}}{dt}=-\alpha{. V}_{n} . \phi_{cells} . V_{o} . \frac{C_{o}}{K_{o}+{\gamma C}_{o}}$ | (9) |
| --- | --- |
| $\frac{dC_{g}}{dt}=-\beta{. V}_{n} . \phi_{cells} . V_{g} . \frac{C_{g}}{K_{g}+C_{g}}$ | (10) |
| $\frac{dC_{la}}{dt}=2 {. V}_{n} . \phi_{cells} . V_{g} . \frac{C_{g}}{K_{g}+C_{g}}$ | (11) |
| $pH=7.4-0.0406C_{la}$ | (12) |
|  |  |

*Table 1: Overview of all parameter values used in this study*

| Parameter | Value | Reference |
| --- | --- | --- |
| *A* | 1.8e-17 | (Mehrian et al. 2017) |
|  | 238 | (Mehrian et al. 2017) |
|  | 341451 | (Mehrian et al. 2017) |
| γ | 0.4514 | (Mehrian et al. 2017) |
|  | 0.6892 | (Mehrian et al. 2017) |
| *s_1_* | -3e-5 | (Mehrian et al. 2017) |
| *s_2_* | 1.5e-4 | (Mehrian et al. 2017) |
| *c_1_* | 20 | (Mehrian et al. 2017) |
| *c_2_* | -250 | (Mehrian et al. 2017) |
| *T_1_* | 60 | (Mehrian et al. 2017) |
| *T_2_* | 70 | (Mehrian et al. 2017) |
| *a_1_* | 0.01 | (Chapman et al. 2014) |
| *a_2_* | 0.03 | (Chapman et al. 2014) |
| *a_3_* | 0.05 | (Chapman et al. 2014) |
| *V_o_* | 1.09e-17 mol/cell/s | (Lambrechts et al. 2014) |
| *V_g_* | 9.5e-17 mol/cell/s | (Zhou et al. 2013) |
| *K_o_* | 1.82e-3 mM | (Carlier et al. 2014) |
| *K_Vn_* | ${10}^{-8}$ | (Mehrian et al. 2017) |
| *K_g_* | 0.3 mM | (Hossain et al. 2015) |
| *φ_cells_* | 2.5e13 cells/m^3^ | (Guyot 2015) |

***Initial conditions***

At the start of the bioreactor culture, 2e^5^ cells were (manually) drop-seeded onto the scaffold. For the numerical analysis, initial seeding was assumed to be homogeneous throughout the entire scaffold. This initial layer was assumed to be $20 \mu m$ based on the reported diameters of MSCs in 2D culture (Darling and Guilak 2008). Assuming a cell seeding efficiency of 60% (experimental evaluation is ongoing), the seeded cell amount distributed into the initial monolayer of $20 \mu m$ thick resulted in an initial cell density $\varphi_{cell}$ of approximately 2.5e^13^ cells/m3 for the scaffolds used in the bioreactor experiments. The value was assumed not to change over time and was used for all scaffold designs.

***Model Implementation***

The reduced model was implemented in MATLAB (MathWorks^®^). The capacity of predicting the evolution of the neotissue growth was tested on five scaffolds with triply periodic internal structures.

***References of the supplementary methods***

Carlier A, van Gastel N, Geris L, Carmeliet G, Van Oosterwyck H. 2014. Size does matter: an integrative in vivo-in silico approach for the treatment of critical size bone defects. PLoS Comput Biol 10(11):e1003888.

Chapman LA, Shipley RJ, Whiteley JP, Ellis MJ, Byrne HM, Waters SL. 2014. Optimising cell aggregate expansion in a perfused hollow fibre bioreactor via mathematical modelling. Plos one 9(8):e105813.

Darling EM, Guilak F. 2008. A neural network model for cell classification based on single-cell biomechanical properties. Tissue Engineering Part A 14(9):1507-1515.

Guyot Y. 2015. A multiphysics multiscale computational framework for the simulation of perfusion bioreactor processes in bone tissue engineering [PhD thesis]: Université de Liège. 99-119 p.

Guyot Y, Papantoniou I, Luyten F, Geris L. 2016. Coupling curvature-dependent and shear stress-stimulated neotissue growth in dynamic bioreactor cultures: a 3D computational model of a complete scaffold. Biomechanics and modeling in mechanobiology 15(1):169-180.

Hossain MS, Bergstrom D, Chen X. 2015. Prediction of cell growth rate over scaffold strands inside a perfusion bioreactor. Biomechanics and modeling in mechanobiology 14(2):333-344.

Lambrechts T, Papantoniou I, Sonnaert M, Schrooten J, Aerts JM. 2014. Model‐based cell number quantification using online single‐oxygen sensor data for tissue engineering perfusion bioreactors. Biotechnology and bioengineering 111(10):1982-1992.

Mehrian M, Guyot Y, papantoniou I, Olofsson S, Sonnaert M, Misener R, Geris L. 2017. Maximizing neotissue growth kinetics in a perfusion bioreactor: an in silico strategy using model reduction and Bayesian optimization Biotechnology and Bioengineering.

Wuertz K, Godburn K, Iatridis JC. 2009. MSC response to pH levels found in degenerating intervertebral discs. Biochemical and biophysical research communications 379(4):824-829.

Zhou X, Holsbeeks I, Impens S, Sonnaert M, Bloemen V, Luyten F, Schrooten J. 2013. Noninvasive real-time monitoring by AlamarBlue® during in vitro culture of three-dimensional tissue-engineered bone constructs. Tissue Engineering Part C: Methods 19(9):720-729.
